# Association between the XRCC1 Polymorphisms and Glioma Risk: A Meta-Analysis of Case-Control Studies

**DOI:** 10.1371/journal.pone.0055597

**Published:** 2013-01-30

**Authors:** Lei Jiang, Xiao Fang, Yi Bao, Jue-Yu Zhou, Xiao-Yan Shen, Mao-Hua Ding, Yi Chen, Guo-Han Hu, Yi-Cheng Lu

**Affiliations:** 1 Department of Neurosurgery, Changzheng Hospital, Second Military Medical University, Shanghai, China; 2 Department of Endocrinology, Changzheng Hospital, Second Military Medical University, Shanghai, China; 3 Institute of Genetic Engineering, Southern Medical University, Guangzhou, China; Baylor College of Medicine, United States of America

## Abstract

**Background:**

X-ray repair cross-complementing group 1 (XRCC1) is one of the DNA repair genes encoding a scaffolding protein that participate in base excision repair (BER) pathway. However, studies on the association between polymorphisms in this gene and glioma have yielded conflicting results. This meta-analysis was performed to derive a more precise estimation between XRCC1 polymorphisms (Arg399Gln, Arg194Trp, and Arg280His) and glioma risk.

**Methods:**

Data were collected from several electronic databases, with the last search up to November 28, 2012. Meta-analysis was performed by critically reviewing 9 studies for Arg399Gln polymorphism (3146 cases and 4296 controls), 4 studies for Arg194Trp polymorphism (2557 cases and 4347 controls), and 4 studies for Arg280His polymorphism (1936 cases and 2895 controls). All of the statistical analyses were performed using the software programs STATA (version 11.0).

**Results:**

The combined results showed that Arg399Gln polymorphism was significantly associated with glioma risk (Gln/Gln versus Arg/Arg: OR = 1.52, 95% CI = 1.03–2.23; recessive model: OR = 1.32, 95% CI = 1.01–1.73; additive model: OR = 1.21, 95% CI = 1.00–1.47), whereas Arg194Trp/Arg280His polymorphisms were all not significantly associated with glioma risk. As for ethnicity, Arg399Gln polymorphism was associated with increased risk of glioma among Asians (Gln/Gln versus Arg/Arg: OR = 1.78, 95% CI = 1.29–2.47; Arg/Gln versus Arg/Arg: OR = 1.28, 95% CI = 1.05–1.56; recessive model: OR = 1.59, 95% CI = 1.16–2.17; dominant model: OR = 1.36, 95% CI = 1.13–1.65; additive model: OR = 1.32, 95% CI = 1.15–1.52), but not among Caucasians. Stratified analyses by histological subtype indicated that the Gln allele of Arg399Gln polymorphism showed borderline association with the risk of glioblastoma among Caucasians. However, no evidence was observed in subgroup analyses for Arg194Trp/Arg280His polymorphisms.

**Conclusions:**

Our meta-analysis suggested that Arg399Gln polymorphism was associated with increased risk of glioma among Asians and borderline increased risk for glioblastoma among Caucasians, whereas Arg194Trp/Arg280His polymorphisms might have no influence on the susceptibility of glioma in different ethnicities.

## Introduction

Gliomas are the most common type of primary brain tumors, including astrocytomas, oligodendrogliomas, oligoastrocytomas, and glioblastoma, which can be classified into four clinical grades (I, II, III and IV) based on histopathological characteristics and biological behaviors. Among them, the glioblastoma (GBM) is the most frequent and malignant histologic type with worse prognosis [1], [2]. Although the etiology of glioma is largely unknown, prior studies have shown that ionizing radiation may be the only established environmental risk factor for glioma. However, not all of those who have been exposed to ionizing radiation will develop glioma, suggesting that other causes, including genetic susceptibility, might play a pivotal role in modifying the risk of glioma.

DNA damage or reduced DNA repair capacity is viewed as an important mechanism in genetic instability and carcinogenesis caused by ionizing radiation and environmental chemical agents. There are four major DNA repair pathways, including base excision repair (BER), nucleotide excision repair (NER), mismatch repair (MMR) and double strand break repair (DSBR) [3]. X-ray repair cross-complementing group 1 (XRCC1) is one of the DNA repair genes encoding a scaffolding protein that participate in BER pathway [4]. It functions as a facilitator or coordinator in BER pathway by directly interacting with poly (ADP-ribose) polymerase (PARP), DNA polymerase beta, and DNA ligase III [4], [5], [6]. Several nonsynonymous coding polymorphisms were identified in this gene, and three of which are most widely investigated including codon 194 (C to T, Arg to Trp), codon 280 (G to A, Arg to His), and codon 399 (G to A, Arg to Gln) [7]. These polymorphisms, involving amino acid change at evolutionarily conserved sequences, may affect DNA repair capacity by changing interactions between XRCC1 protein and other base excision repair proteins. Considering the importance of XRCC1 in BER pathway and the potential influence of genetic variants in this gene on the repair capacity for DNA damage, a large number of studies were conducted to investigate the association between these three XRCC1 polymorphisms and cancer risk in humans [8], [9], [10], [11], [12], [13], [14]. Although several epidemiological studies have also assessed the relationship between these polymorphisms and the risk of glioma [15], [16], [17], [18], [19], [20], [21], [22], [23], [24], [25], [26], [27], [28], the results are to some extent divergent, but nevertheless intriguing, which may be due to limitations in individual studies. So far, no quantitative summary of the evidence has ever been performed. To gain better insight into the impact of these polymorphic variants on the risk of glioma, we performed a meta-analysis with subgroup analysis from all published case–control studies.

## Materials and Methods

### Study identification and selection

A comprehensive literature search was performed using PubMed and EMBASE to identify studies that evaluated the association between XRCC1 polymorphisms and the risk of glioma up to November 28, 2012 with the following terms and keywords: (XRCC1 or ‘X-ray repair cross-complementation group 1’ or ‘DNA repair gene’) and (polymorphism or variant or variation) and (glioma or ‘brain tumor’). The search was limited to human studies. Additional studies were identified by hand searching references in original articles and review articles. The following criteria were used for the study selection: 1) a case–control study evaluating at least one polymorphism in the XRCC1 gene; 2) studies with full text articles; 3) sufficient data for estimating an odds ratio (OR) with 95% confidence interval (CI); 4) genotype distribution of control population must be in Hardy–Weinberg equilibrium (HWE); 5) no overlapping data. If studies had the same or overlapping data, only the largest study should be included in the final analysis.

### Data extraction

Information was carefully extracted from all the eligible studies independently by three investigators according to the selection criteria listed above. The following data were collected from each study: first author, publication year, country, racial descent (categorized as Asian, Caucasian, or mixed descent), source of controls, genotyping method, numbers of cases and controls, genotype frequency of cases and controls, and the result of Hardy–Weinberg equilibrium test. For conflicting evaluation, a consensus was reached by discussion. We did not define any minimum number of patients for inclusion in our meta-analysis.

### Statistical analysis

A Chi-square test using a web-based program (http://ihg2.helmholtz-muenchen.de/cgi-bin/hw/hwa1.pl) was applied to determine if observed frequencies of genotypes in controls conformed to HWE (*P*<0.05 was considered significant). Crude ORs together with their corresponding 95% CIs were used to assess the strength of association between these three XRCC1 polymorphisms (Arg399Gln, Arg194Trp, and Arg280His) and glioma risk for each study. The pooled ORs were performed for additive model (a allele versus A allele, a was for the minor allele and A was for the major allele), codominant model (aa versus AA, Aa versus AA), dominant model (aa+Aa versus AA), recessive model (aa versus Aa+AA) respectively. Heterogeneity assumption was checked by a chi-square-based *Q* test [29], and *I* ^2^ statistics was calculated to quantify the proportion of the total variation across studies due to heterogeneity [30]. A *p*-value of >0.05 for the *Q*-test indicated a lack of heterogeneity among studies, so that the pooled OR estimate of each study was calculated by the fixed-effects model (the Mantel–Haenszel method) [31]. Otherwise, the random-effects model (the DerSimonian and Laird method) was used [32]. Subgroup analyses were performed by ethnicity, source of controls and histological subtype (glioblastoma). Sensitivity analysis was mainly performed by sequential omission of individual studies. An estimate of potential publication bias was carried out by the funnel plot, in which the standard error of log (OR) of each study was plotted against its log (OR). Funnel plot asymmetry was further assessed by the method of Egger's linear regression test (P<0.05 was considered a significant publication bias) [33]. All of the statistical analyses were performed using STATA version 11.0 (Stata, College Station, TX, USA).

## Results

### Extraction process and study characteristics

**Figure 1:**
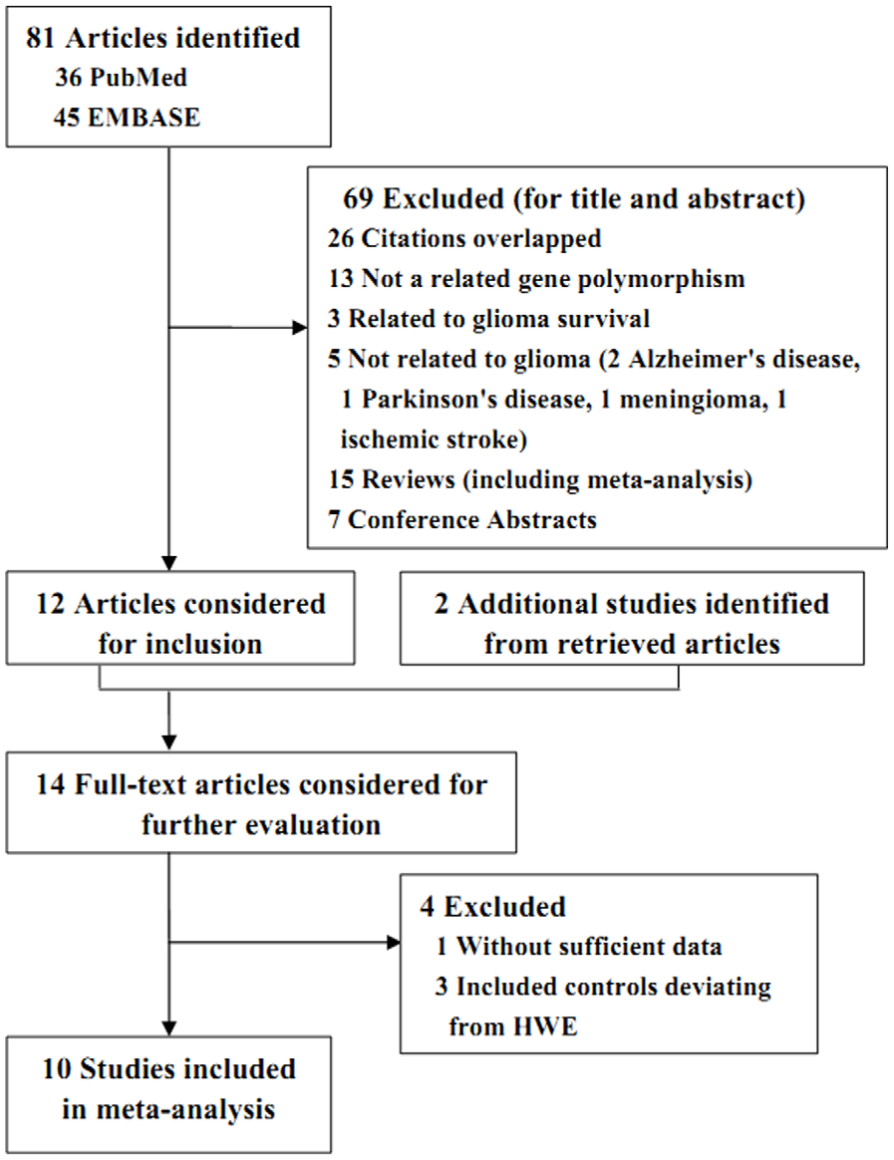
Flow of Included Studies.

Based on our search criterion, 81 articles were found, but only 14 full-text articles [15], [16], [17], [18], [19], [20], [21], [22], [23], [24], [25], [26], [27], [28] were preliminarily identified for further detailed evaluation (Figure 1). Among them, one studies [25] were excluded because the data of genotyping distribution were missing and three studies [26], [27], [28] were excluded because of included controls deviating from HWE. Thus, a total of 10 studies [15], [16], [17], [18], [19], [20], [21], [22], [23], [24] were eligible for the meta-analysis. Nevertheless, of these eligible studies, the study by McKean-Cowdin et al. [18] combined the genetic data for XRCC1 polymorphisms (including Arg194Trp, Arg399Gln) from four centers in the United States that have conducted case-control studies on glioblastoma multiforme, including the National Cancer Institute (NCI), the National Institute for Occupational Safety and Health (NIOSH), the University of Texas M. D. Anderson Cancer Center (MDA), and the University of California at San Francisco (UCSF). And the other three studies by Rajaraman et al. [17] (including Arg399Gln, Arg194Trp, and Arg280His), Liu et al. [19] (including Arg399Gln, Arg194Trp) and Felini et al. [22] (including Arg399Gln) were reported from NCI, MDA and UCSF, respectively. Actually, these three studies may contain partial overlapping data with the study by McKean-Cowdin et al. [18] when carefully reading the full texts, and only the larger study should be selected for the analysis. For this, the studies by Rajaraman et al., Liu et al. and Felini et al. were selected for Arg399Gln polymorphism, and the study by McKean-Cowdin et al. was eligible for Arg194Trp polymorphism. Therefore, there were 9 studies for Arg399Gln polymorphism (3146 cases and 4296 controls), 4 studies for Arg194Trp polymorphism (2557 cases and 4347 controls), and 4 studies for Arg280His polymorphism (1936 cases and 2895 controls). The main characteristics of these included studies were summarized in Table 1. There were 8 studies of Caucasians [16], [17], [18], [19], [20], [21], [22], [23] and two studies of Asians [15], [24]. Two studies [18], [20] provided the genotype data of high-grade glioma (glioblastoma).

**Table 1:** Characteristics of studies included in the meta-analysis.

Author	Year	Ethnicity	Country	SNPs studied	Genotyping	Control source	Cases/Controls	MAF	HWE
Wang	2004	Caucasian	USA	399	PCR-RFLP	HB	309/342	0.38	0.92
Felini	2007	Caucasian	USA	399	PCR-RFLP	PB	366/427	0.35	0.83
Kiuru	2008	Caucasian	European countries	399, 194, 280	PCR-RFLP	PB	699/1549, 700/1556,701/1560	0.35, 0.06, 0.05	0.17, 0.13, 0.85
Cengiz	2008	Caucasian	Turkey	399	PCR-RFLP	HB	35/87	0.27	0.07
Liu	2009	Caucasian	USA	399, 194	MassARRAY	PB	373/364, 210/365	0.34, 0.08	0.05, 0.62
McKean-Cowdin	2009	Caucasian	USA	399, 194	TaqMan	Mixed	1003/1971, 962/1922	0.35, 0.07	0.09, 0.27
Rajaraman	2010	Caucasian	USA	399, 194, 280	TaqMan	HB	350/478, 342/468, 340/466	0.36, 0.08, 0.05	0.05, 0.21, 0.75
Yosunkaya	2010	Caucasian	Turkey	399	PCR-RFLP	HB	119/180	0.30	0.45
Zhou	2011	Asian	China	399, 194, 280	PCR-RFLP	HB	271/289, 271/289, 271/289	0.29, 0.25, 0.09	0.96, 0.14, 0.09
Wang	2012	Asian	China	399, 194, 280	PCR-RFLP	HB	624/580, 624/580, 624/580	0.28, 0.21, 0.10	0.74, 0.14, 0.14

Abbreviations: HWE, Hardy-Weinberg equilibrium; PB, population-based; HB, hospital-based; RFLP, restriction fragment length polymorphism;

TaqMan, real-time TaqMan analysis; MassARRAY, genotyping was performed using the Sequenom MassARRAY platform

### Meta-analysis results

**Figure 2:**
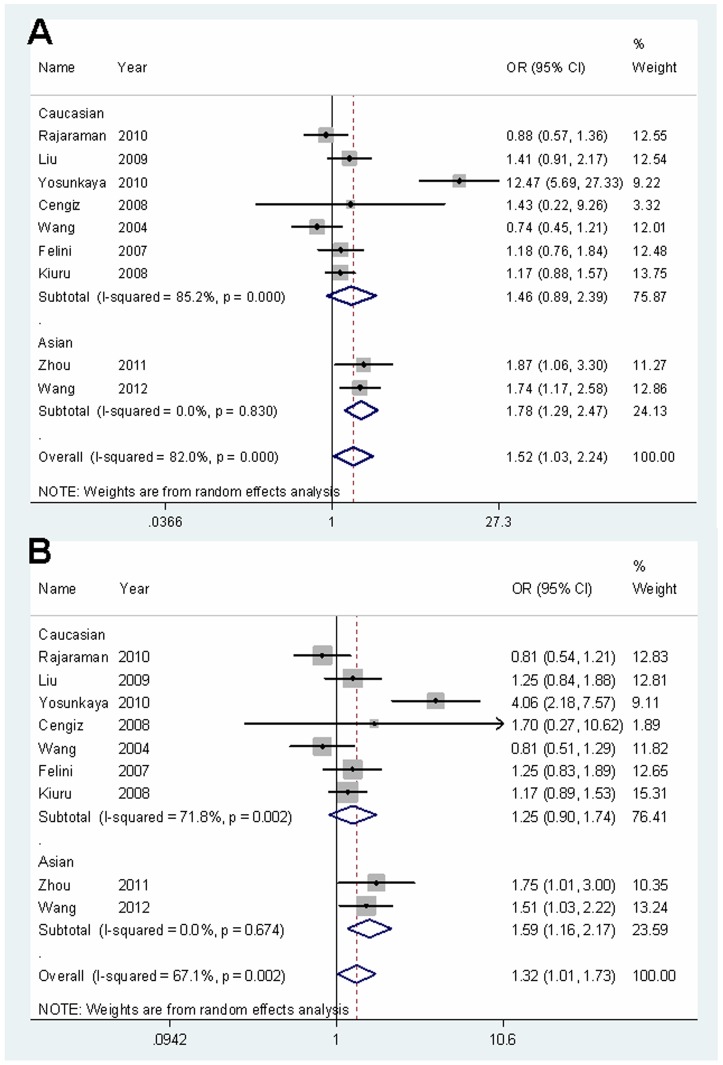
Forest plots of ORs with 95% CI for XRCC1 Arg399Gln polymorphism and the risk of glioma observed in subgroup analyses by ethnicity (random effects). The center of each square represents the OR, the area of the square is the number of sample and thus the weight used in the meta-analysis, and the horizontal line indicates the 95%CI. (A) Gln/Gln versus Arg/Arg. (B) Recessive model.

Table 2 listed the main results of the meta-analysis for XRCC1 polymorphisms. For Arg399Gln polymorphism, significant association between this polymorphism and glioma risk was observed when all eligible studies were pooled into meta-analysis (Gln/Gln versus Arg/Arg: OR = 1.52, 95% CI = 1.03–2.23; recessive model: OR = 1.32, 95% CI = 1.01–1.73; additive model: OR = 1.21, 95% CI = 1.00–1.47; Table 2). As for ethnicity, our results showed Arg399Gln polymorphism was associated with increased risk of glioma among Asians (Gln/Gln versus Arg/Arg: OR = 1.78, 95% CI = 1.29–2.47; Arg/Gln versus Arg/Arg: OR = 1.28, 95% CI = 1.05–1.56; recessive model: OR = 1.59, 95% CI = 1.16–2.17; dominant model: OR = 1.36, 95% CI = 1.13–1.65; additive model: OR = 1.32, 95% CI = 1.15–1.52; Table 2, Figure 2), but not among Caucasians. When stratified by the source of controls, we found a borderline significant increased risk of glioma in population-based studies among Caucasians (Gln/Gln versus Arg/Arg: OR = 1.23, 95% CI = 1.00–1.52), but not in hospital-based studies among Caucasians (Table 2). Similarly, the Gln allele of Arg399Gln polymorphism showed borderline association with the risk of glioblastoma among Caucasians (additive model: OR = 1.10, 95% CI = 1.00–1.21).

**Table 2:** Results of meta-analysis for Arg399Gln, Arg194Trp and Arg280His polymorphisms and the risk of glioma and glioblastoma.

Genetic model		Recessive model			Dominant model			Homozygote			Heterozygote			Additive model		
Arg399Gln	n	Gln/Gln vs. Arg/Gln + Arg/Arg			Gln/Gln + Arg/Gln vs. Arg/Arg			Gln/Gln vs. Arg/Arg			Arg/Gln vs. Arg/Arg			Gln vs. Arg		
		OR(95%CI)	*P*_h_	*I* ^2^(%)	OR(95%CI)	*P*_h_	*I* ^2^(%)	OR(95%CI)	*P*_h_	*I* ^2^(%)	OR(95%CI)	*P*_h_	*I* ^2^(%)	OR(95%CI)	*P*_h_	*I* ^2^(%)
Total	9(3146/4296)	1.32(1.01–1.73)	0.002	67.1	1.25(0.98–1.60)	0.000	82.8	1.52(1.03–2.23)	0.000	82.0	1.19(0.95–1.50)	0.000	77.4	1.21(1.00–1.47)	0.000	85.4
Ethnicity																
Caucasian	7(2251/3427)	1.25(0.90–1.74)	0.002	71.8	1.24(0.90–1.71)	0.000	85.9	1.46(0.89–2.39)	0.000	85.2	1.19(0.88–1.60)	0.000	81.8	1.19(0.92–1.53)	0.000	88.1
Asian	2(895/869)	1.59(1.16–2.17)	0.674	0.0	1.36(1.13–1.65)	0.660	0.0	1.78(1.29–2.47)	0.830	0.0	1.28(1.05–1.56)	0.524	0.0	1.32(1.15–1.52)	0.918	0.0
Source of controls																
PB	3(1438/2340)	1.21(0.99–1.47)	0.944	0.0	1.07(0.94–1.23)	0.302	16.5	1.23(1.00–1.52)	0.780	0.0	1.03(0.89–1.19)	0.290	19.1	1.09(0.99–1.20)	0.452	0.0
HB	6(1708/1956)	1.44(0.89–2.34)	0.000	79.1	1.39(0.91–2.11)	0.000	88.0	1.79(0.89–3.59)	0.000	88.3	1.32(0.90–1.92)	0.000	83.4	1.29(0.92–1.80)	0.000	90.1
HB*	4(813/1087)	1.39(0.61–3.13)	0.000	85.7	1.45(0.66–3.17)	0.000	92.5	1.83(0.53–6.26)	0.000	92.5	1.39(0.68–2.82)	0.000	89.8	1.27(0.70–2.31)	0.000	93.8
Histological subtype																
Glioblastoma	2(1323/3520)	1.13(0.94–1.36)	0.660	0.0	1.13(0.99–1.28)	0.721	0.0	1.19(0.98–1.46)	0.817	0.0	1.11(0.96–1.27)	0.612	0.0	1.10(1.00–1.21)	0.943	0.0

Arg194Trp	n	Trp/Trp vs. Arg/Trp + Arg/Arg			Trp/Trp + Arg/Trp vs. Arg/Arg			Trp/Trp vs. Arg/Arg			Arg/Trp vs. Arg/Arg			Trp vs. Arg		
		OR(95%CI)	*P*_h_	*I* ^2^(%)	OR(95%CI)	*P*_h_	*I* ^2^(%)	OR(95%CI)	*P*_h_	*I* ^2^(%)	OR(95%CI)	*P*_h_	*I* ^2^(%)	OR(95%CI)	*P*_h_	*I* ^2^(%)
Total	4(2557/4347)	1.35(0.88–2.06)	0.719	0.0	0.98(0.86–1.11)	0.792	0.0	1.36(0.89–2.09)	0.733	0.0	0.90(0.75–1.08)	0.838	0.0	1.00(0.89–1.13)	0.733	0.0
Ethnicity																
Caucasian	2(1662/3478)	1.55(0.54–4.47)	0.296	8.5	0.92(0.76–1.10)	0.954	0.0	1.54(0.53–4.42)	0.297	8.0	0.90(0.75–1.08)	0.838	0.0	0.93(0.78–1.11)	0.923	0.0
Asian	2(895/869)	1.32(0.83–2.09)	0.683	0.0	1.05(0.87–1.27)	0.919	0.0	1.33(0.83–2.12)	0.716	0.0	1.02(0.84–1.24)	0.835	0.0	1.07(0.91–1.25)	0.952	0.0
Source of controls																
PB	2(910/1921)	1.58(0.43–5.82)	0.232	30.0	0.92(0.72–1.17)	0.910	0.0	1.56(0.42–5.77)	0.234	29.3	0.90(0.70–1.16)	0.770	0.0	0.94(0.74–1.19)	0.940	0.0
HB	3(1237/1337)	1.28(0.81–2.02)	0.748	0.0	0.97(0.81–1.15)	0.153	46.7	1.29(0.81–2.05)	0.743	0.0	0.94(0.79–1.13)	0.214	35.1	1.00(0.87–1.16)	0.117	53.3
Histological subtype																
Glioblastoma	2(1282/3478)	1.21(0.36–4.02)	0.528	0.0	0.96(0.79–1.17)	0.501	0.0	1.20(0.36–3.99)	0.520	0.0	0.95(0.78–1.16)	0.543	0.0	0.97(0.80–1.17)	0.471	0.0

Arg280His	n	His/His vs. Arg/His + Arg/Arg			His/His + Arg/His vs. Arg/Arg			His/His vs. Arg/Arg			Arg/His vs. Arg/Arg			His vs. Arg		
		OR(95%CI)	*P*_h_	*I* ^2^(%)	OR(95%CI)	*P*_h_	*I* ^2^(%)	OR(95%CI)	*P*_h_	*I* ^2^(%)	OR(95%CI)	*P*_h_	*I* ^2^(%)	OR(95%CI)	*P*_h_	*I* ^2^(%)
Total	4(1936/2895)	0.73(0.35–1.50)	0.255	26.1	0.98(0.82–1.17)	0.569	0.0	0.74(0.36–1.52)	0.252	26.6	1.00(0.83–1.19)	0.620	0.0	0.97(0.82–1.14)	0.458	23.0
Ethnicity																
Caucasian	2(1041/2026)	0.52(0.09–3.19)	0.920	0.0	0.88(0.68–1.14)	0.491	0.0	0.52(0.08–3.16)	0.913	0.0	0.89(0.69–1.16)	0.516	0.0	0.88(0.69–1.12)	0.475	0.0
Asian	2(895/869)	0.78(0.36–1.73)	0.050	74.0	1.08(0.85–1.37)	0.586	0.0	0.80(0.36–1.76)	0.050	74.0	1.11(0.86–1.42)	0.925	0.0	1.05(0.84–1.30)	0.314	1.4
Source of controls																
PB	1(701/1560)	0.56(0.06–4.98)	–	–	0.93(0.69–1.26)	–	–	0.55(0.06–4.95)	–	–	0.94(0.70–1.27)	–	–	0.93(0.69–1.24)	–	–
HB	3(1235/1335)	0.76(0.35–1.63)	0.137	49.6	1.01(0.81–1.25)	0.397	0.0	0.77(0.36–1.65)	0.136	49.9	1.03(0.82–1.28)	0.457	0.0	0.99(0.81–1.20)	0.290	19.2
Histological subtype																
Glioblastoma	1(320/1560)	0.547(0.03–10.05)	–	–	0.80(0.52–1.23)	–	–	0.53(0.03–9.87)	–	–	0.82(0.54–1.26)	–	–	0.79(0.52–1.20)	–	–

*P*_h_ *P* values for heterogeneity from *Q* test. Random-effects model was used when *P* value for heterogeneity test <0.05; otherwise, fixed-model was used. HB* hospital-based studies among Caucasians.

As for Arg194Trp/Arg280His polymorphisms, the combined results based on all studies did not showed any association between Arg194Trp/Arg280His polymorphisms and glioma risk for all genetic models (Table 2). Furthermore, there was no evidence for the association between each polymorphism and glioma risk in subgroup analyses based on the source of controls, ethnicity and histological subtype (Table 2).

### Test of heterogeneity and sensitivity analyses

**Figure 3:**
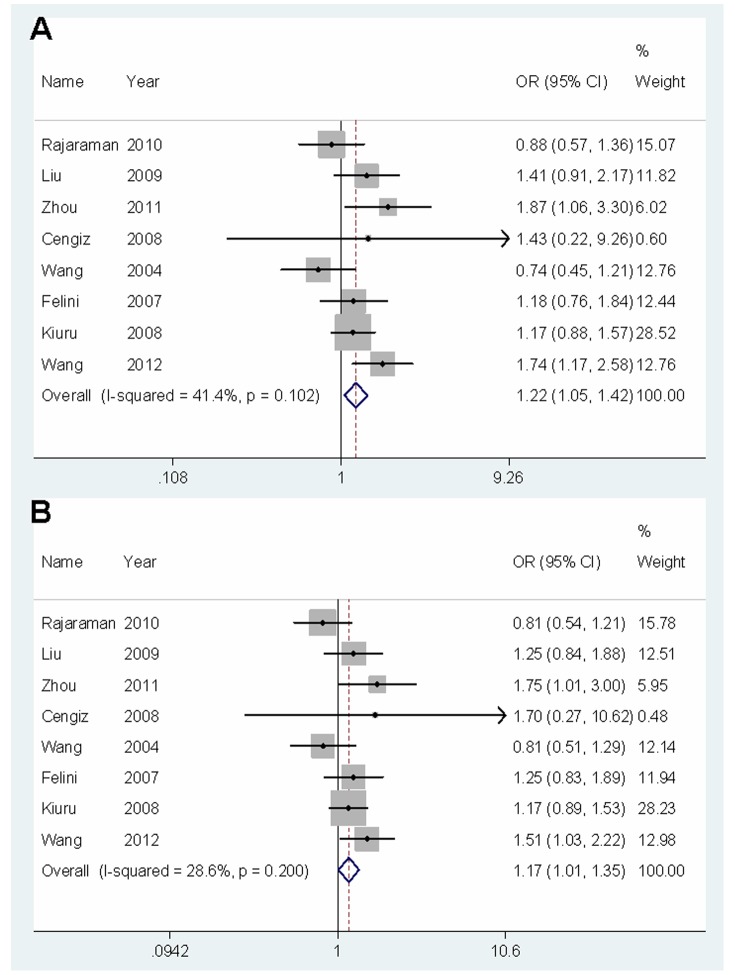
Forest plot of ORs with 95% CI for glioma risk associated with the Arg399Gln polymorphism (random effects) after exclusion of the study contributing to substantial heterogeneity. The center of each square represents the OR, the area of the square is the number of sample and thus the weight used in the meta-analysis, and the horizontal line indicates the 95%CI. (A) Gln/Gln versus Arg/Arg. (B) Recessive model.

The heterogeneity test showed that there was no significant between-study heterogeneity in terms of the XRCC1 Arg194Trp/Arg280His polymorphisms, but significant heterogeneity for the Arg399Gln polymorphism in overall comparisons (Table 2). To explore the potential sources of heterogeneity across studies, we assessed the pooled ORs under all comparisons via stratification and sensitivity analyses. As a result, the study by Yosunkaya et al. [16] was found to contribute to substantial heterogeneity because it was significantly decreased under all comparisons after exclusion of this study (Figure 3). Furthermore, influence analysis was performed to assess the influence of each individual study on the pooled ORs by sequential omission of individual studies. The results suggested that the study by Yosunkaya et al. did not significantly affect the pooled ORs for the Arg399Gln polymorphism. However, it was likely that the studies for Asian population [15], [24] dominated the results for Arg399Gln polymorphism in the total population, since once we omitted one of these studies, the results showed that this polymorphism was no longer associated with the risk of glioma in homozygote comparison, recessive model and additive model. Additionally, the corresponding pooled ORs for Arg194Trp/Arg280His polymorphisms were not materially altered by removing any individual study (data not shown).

### Publication bias

**Figure 4:**
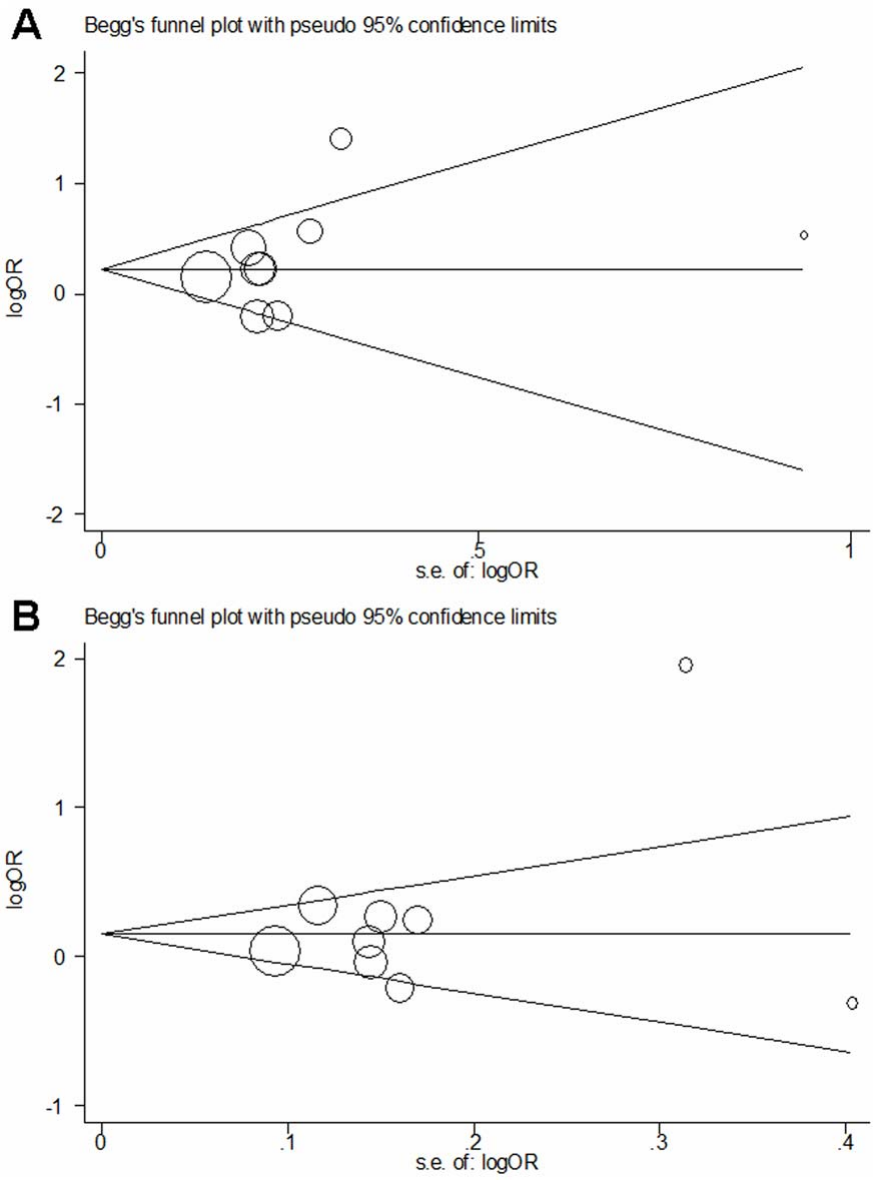
Begg's funnel plots of Arg399Gln polymorphism and glioma risk for publication bias test. Each point represents a separate study for the indicated association. Log (OR), natural logarithm of OR. Horizontal line, mean effect size. (A) Recessive model. (B) Dominant model.

Both Begg's funnel plot and Egger's test were performed to assess the publication bias of literatures. All these three genetic polymorphisms showed consistent results, indicating no publication biases. Take the Arg399Gln polymorphism as an example. The shapes of the funnel plot did not indicate any evidence of obvious asymmetry in both recessive model and dominant model (Figure 4), and the Egger's test suggested the absence of publication bias (P = 0.383 for Arg/Gln versus Arg/Arg, P = 0.276 for Gln/Gln versus Arg/Arg, P = 0.347 for dominant model and P = 0.338 for recessive model, P = 0.422 for additive model, respectively).

## Discussion

DNA disruptions can lead to gene rearrangements, translocations, amplifications, and deletions, which can in turn contribute to cancer development [3]. The mechanisms for repairing DNA damaged by chemicals or radiation are varied and complicated, which play a critical role in maintaining genome integrity and preventing carcinogenesis [34]. Until now, it has been reported that more than a hundred proteins implicated in DNA repair pathways in human cells. Genetic variations in corresponding DNA repair genes are thought to modify DNA repair capacity and suggested to be related to cancer risk [35]. The XRCC1 is located on chromosome 19q13.2–13.3 and is 33 kb in length, encoding a scaffolding protein in BER pathway that functions in the repair of single-strand breaks, which is the most common lesion of cellular DNA injury. Three coding polymorphisms (Arg399Gln, Arg194Trp, and Arg280His) were most extensively studied in the XRCC1 gene, and it was widely accepted that functional variants in this gene may play a crucial role in the development of cancer because of the alteration of base excision repair functions [36]. To date, many studies have investigated the associations between XRCC1 variants in DNA repair genes and the risk of glioma in different populations, but the results remain contradictory [15], [16], [17], [18], [19], [20], [21], [22], [23], [24], [25], [26], [27], [28]. The individual studies might have been underpowered to detect the overall effect of polymorphisms on the risk of glioma. In order to avoid this issue, we performed a meta-analysis to provide the most comprehensive assessment of the association between XRCC1 polymorphisms and glioma risk.

The combined results based on all studies showed that Arg399Gln polymorphism was significantly associated with glioma risk, whereas Arg194Trp/Arg280His polymorphisms were all not significantly associated with glioma risk. As for ethnicity, Arg399Gln polymorphism was associated with increased risk of glioma among Asians in all genetic models, but not among Caucasians. So the significant results for Arg399Gln polymorphism in the total population might be due to the influence of the studies for Asians. When stratified by the source of controls, our results found little evidence of an association between XRCC1 Arg399Gln polymorphism and increased risk of glioma among Caucasians using population-based controls but not using hospital-based controls, indicating the importance of the use of proper and representative cancer-free control subjects because the allele distribution in hospital-based controls may not be very representative of the general population. Besides, individuals carrying the Gln allele may have a borderline increased cancer risk for glioblastoma among Caucasians. There was no evidence for the association between Arg194Trp/Arg280His polymorphisms and glioma risk in subgroup analyses based on ethnicity, source of controls and histological subtype. Nevertheless, considering the limited studies of the Arg194Trp and Arg280His polymorphisms, our results related to these two polymorphisms should always be treated as preliminary. Additionally, our results for subgroup analyses should be interpreted in a conservative manner and need further validation in larger well-designed studies.

Previous studies have suggested that Arg399Gln polymorphism is significantly associated with increased risk of glioma. For example, Yosunkaya et al. [16] reported that 399Gln allele carries a 3.5 times greater risk for glioma. Similarly, Liu et al. [19] provided evidence that compared with wild-type homozygote carriers, significant increased risk effects were associated with XRCC1 Arg399Gln variants (adjusted OR, 1.43; 95% CI, 1.05–1.92), which were more pronounced in patients with high-grade gliomas (adjusted OR, 1.46; 95% CI, 1.01–2.08). Meanwhile, the studies for Asian population [15], [24], [26] uniformly showed that individuals with the Gln/Gln genotype had a significantly increased likelihood of developing glioma among Chinese population, which agreed with our conclusion. However, many other studies did not find any statistically significant association between Arg399Gln polymorphism and the risk of glioma in Caucasian population [17], [18], [20], [21], [22], [23], [25], [27], which was also in line with our results for Caucasians. As for the other two polymorphisms, we also did not find significant association of polymorphisms in Arg194Trp and Arg280His with the risk of glioma, which were consistent with the majority but not all previous studies [15], [18], [19], [20], [24]. The studies by Custodio et al. [27] and Hu et al. [26] found similar results in glioma that increased risk was observed in individuals with 194Trp allele. Whereas decreased glioma risk was associated with the Arg194Trp polymorphism in dominant model (OR = 0.65, 95% CI = 0.42–0.99) [17]. The inconsistency of these studies may be explained by differences in population background, source of controls, sample size, and also by chance. In fact, differences in the allele frequencies of these three polymorphisms in Asians and Caucasians have been reported [37], [38].

Interestingly, previous meta-analyses also have confirmed that Arg399Gln polymorphism is associated with the risk of childhood ALL [9], cervical cancer [39], breast cancer [40], [41], [42], and prostate cancer [43], [44] among Asians, but not among Caucasians, which coincided with our opinion, suggesting that this polymorphism may modify the risk of cancer in different ethnicities. And our results revealed that these three polymorphisms have no influence on the susceptibility of glioma among Caucasians, supported by several published meta-analyses for other cancers, such as colorectal cancer [45], and bladder cancer [46]. On the other hand, the link between Arg194Trp polymorphism and increased cancer risk has been demonstrated in previous meta-analyses that focused on cervical cancer [39], lung cancer [47], [48], [49], esophageal cancer [47], [50], gastric cancer [51] and oral cancer [52]. And two studies [53], [54] provided evidence that Arg280His polymorphism was associated with increased risks of skin cancer and hepatocellular carcinoma, although this polymorphism is not significantly associated with the risk of other cancers in many studies. These studies showed inconsistent conclusions probably due to different roles of these three polymorphisms in different cancers or different ethnicities. Also, the discrepancies might be partially owing to the existence of gene-to-gene or gene-to-environment interactions, or the influence of the genetic variant may be masked by other as-yet-unidentified causal genes involved in carcinogenesis, because the low penetrance genetic effects of single polymorphism usually depends on interaction with other polymorphisms and/or a particular environmental exposure including dietary and lifestyle factors. Another possibility is that studies with small sample sizes may be underpowered for detecting a small but real association. Hence, further confirmation of existing findings is still needed in future studies.

Furthermore, two recent meta-analyses by Wei et al. [55] and Jacobs et al. [56] estimated the association between Arg399Gln polymorphism and glioma risk, which was basically in accordance with our results that Arg399Gln polymorphism may contribute to the susceptibility of glioma in Asians [55], but not in Caucasians [55], [56]. However, the data reported by Wei et al. [55] for the studies by Kiuru et al. [20] and Wang et al. [23] do not seem in line with the data provided in their original publications. The number for Arg/Arg, Arg/Gln, Gln/Gln in controls provided by Kiuru et al. [20] and Wang et al. [23], respectively, are 212–233–74 and 147–147–48. Interestingly enough, after carefully inspecting the original data, the frequencies we have retrieved in controls are 645–728–176 and 131–162–49, respectively. In this meta-analysis, we also found that the study by Liu et al. [19] only provided the total number of variant genotypes was included in the analysis for dominant model, but not for other genetic models. Actually the numbers in cases and controls could be calculated by the minor allele frequency (MAF) provided by Liu et al. [19]. And the separate data for Arg/Arg, Arg/Gln, Gln/Gln in cases and controls, respectively, are 149–162–62 and 169–145–50. Meanwhile, the data for sample sizes reported by Jacobs et al. [56] for the studies by Kiuru et al. [20] and Cengiz et al. [21] also do not seem in line with the data provided in their original publications. The numbers of cases and controls reported by Jacobs et al. [56] for these two studies, respectively, are 1019–1549 and 135–87. In fact, we found that the numbers reported by Kiuru et al. [20] and Cengiz et al. [21] for cases and controls should be 699–1549 and 35–87, respectively. Furthermore, at least two eligible studies [18], [24] were not included in the meta-analysis by Wei et al. [55], while the studies by McKean-Cowdin et al. [18] and Wang et al. [23] were excluded in the meta-analysis by Jacobs et al. [56] for providing duplicate data. However, these two studies do not contain overlapping data when carefully reading the full texts. As mentioned above, the study by McKean-Cowdin et al. [18] combined the genetic data for XRCC1 polymorphisms from four centers (NCI, MDA, UCSF and NIOSH) and contained partial overlapping data with the study by Rajaraman et al. (NCI) [17], Liu et al. (MDA) [19] and Felini et al. (UCSF) [22], respectively. Hence, the ongoing uncertainty still exists and the conclusion by these two meta-analyses was not entirely credible.

Unfortunately, another newly published meta-analysis by Sun et al. [57] likewise did not recognize the aforementioned overlapping data when exploring the association between Arg399Gln/Arg194Trp polymorphisms and glioma risk, which might lead to duplicate counting of subjects and overestimation of intervention effects in meta-analyses [58], [59], [60], because subjects from the same trials are reanalyzed repeatedly, without disclosure, in different studies. For this, researchers should find out the overlapping data in the included studies during a meta-analysis. And the validity of meta-analyses done without looking into this problem is questionable [60]. Moreover, at least two studies [21], [24] were not included in the meta-analysis by Sun et al. [57]. Thus, the ongoing uncertainty still exists and the conclusion by Sun et al. [57] might be biased by the inclusion of overlapping data. In our meta-analysis, we accurately assessed the association between these XRCC1 polymorphisms and the risk of glioma and its histological subtypes by taking into account the effects of overlapping data.

In addition, we have to mention the test of heterogeneity, an important index on the evidence quality of a meta-analysis. Despite some diversity in the studies about designs, sample sizes, inclusion criteria, and ethnicity, significant heterogeneity between studies was only observed for the Arg399Gln polymorphism, but not for the other two polymorphisms. And then stratification and sensitivity analyses were used to explore the sources of heterogeneity. We found that the study by Yosunkaya et al. [16] did contribute to potential heterogeneity, while influence analysis suggested that the pooled ORs for the Arg399Gln polymorphism not be influenced by this study. In view of this, the results of our meta-analysis, in essence, are sound and reliable.

In interpreting our results of the current meta-analysis, some limitations should be acknowledged. First, the number of published studies was not sufficiently large for a comprehensive analysis, especially for stratified analyses by ethnicity and histological subtype. Because of limited available data for Asian population and Arg194Trp/Arg280His polymorphisms, our results should be interpreted with caution. Larger studies are needed to clarify whether these polymorphisms could truly affect the risk of glioma in different ethnicities. Second, lacking the original data for the included studies limited our further evaluation of potential interactions among gene–gene, gene–environment, or even different polymorphism loci of the same gene, which all may affect cancer risk. In fact, the combined effects of various DNA repair gene polymorphisms on cancer risk have already been demonstrated [20], [61], [62]. For instance, carriers of both XRCC1 Gln399Gln and XRCC3 Met241Met were associated with a three-fold increased risk of glioma [20]. Third, our results were based on single-factor estimates without adjustment for other risk factors such as age, gender, smoking status, drinking consumption, environmental factors and other variables, which might have caused serious confounding bias. Several studies have suggested the effect of a possible interaction between XRCC1 polymorphisms and environmental factors on cancer risk [63], [64]. For example, Liu et al. [19] concluded that the increased glioma risk effect of XRCC1 Arg399Gln was more evident in females, while Gln/Gln genotype is associated with a decreased risk of bladder cancer among ever smokers. Last but not the least, some inevitable publication bias might exist in the results because only published studies were retrieved although the funnel plot and Egger's test indicated no remarkable publication bias.

In conclusion, this meta-analysis suggested that Arg399Gln polymorphism was associated with increased risk of glioma among Asians and borderline increased risk for glioblastoma among Caucasians, whereas Arg194Trp/Arg280His polymorphisms might have no influence on the susceptibility of glioma in different ethnicities. Nevertheless, larger population-based and well-designed studies using standardized unbiased genotyping methods are warranted to clarify the effects of gene–gene and gene–environment interactions on these polymorphisms and the risk of glioma and its histological subtypes in specific populations, especially in Asian population. Additional studies exploring the combined effects of these XRCC1 polymorphisms or different polymorphisms in genes involved in DNA repair pathway should be investigated.

## Supporting Information

Table S1 **PRISMA 2009 Checklist for this Meta-analysis.**
(DOC)
